# Novel *SKIC3* variants in tricho-hepato-enteric syndrome with hemochromatosis

**DOI:** 10.1038/s41439-025-00318-y

**Published:** 2025-07-18

**Authors:** Kayo Ochiai, Yoshinori Aoki, Naoshi Yamada, Murasaki Aman, Atsushi Yamashita, Masatoshi Yamaguchi, Daisuke Nakato, Toshiki Takenouchi, Kenjiro Kosaki, Yuki Kodama, Hiroshi Moritake

**Affiliations:** 1 Division of Pediatrics, Faculty of Medicine, University of Miyazaki, Miyazaki, Japan; 2 Perinatal Center, University of Miyazaki Hospital, Miyazaki, Japan; 3 Obstetrics & Gynecology, Faculty of Medicine, University of Miyazaki, Miyazaki, Japan; 4 Division of Pathophysiology, Department of Pathology, Faculty of Medicine, University of Miyazaki, Miyazaki, Japan; 5 Division of Clinical Genetics, University of Miyazaki, Miyazaki, Japan; 6 Center for Medical Genetics, Keio University School of Medicine, Tokyo, Japan; 7 Department of Child Neurology, Okayama University Graduate School of Medicine, Dentistry and Pharmaceutical Sciences, Okayama, Japan

**Keywords:** Genetics research, Disease genetics

## Abstract

Tricho-hepato-enteric syndrome (THES), a rare autosomal recessive disorder caused by variants in the *SKIC3* or *SKIC2* gene, is characterized by intractable diarrhea, woolly hair, growth restriction and liver disease. Here we report a neonatal case of THES with neonatal hemochromatosis, in which the novel compound heterozygous *SKIC3* variants NM_014639.4:c.815_816del p.(Gly272AlafsTer9) and NM_014639.4:c.2284G>A p.(Gly762Arg) were identified. Further research is needed to elucidate the mechanisms underlying iron metabolism dysregulation in THES.

Tricho-hepato-enteric syndrome (THES; OMIM 222470) is a rare autosomal recessive disorder with an estimated global incidence of 1 in 1,000,000 live births^1^. THES is caused by loss of function in either the SKI3 subunit of the superkiller complex (*SKIC3*), also known as *TTC37* (OMIM 614589), or the SKI2 subunit (*SKIC2*), also known as *SKIV2L* (OMIM 600478)^2^. These proteins are components of the human superkiller (SKi) complex, which is involved in exosome-mediated RNA surveillance, including the regulation of normal mRNA and degradation of nonfunctional mRNA^1^. THES is characterized by intractable diarrhea from infancy, characteristic woolly hair, distinctive facial features, intrauterine growth restriction, immune deficiency and liver disease^1^. The phenotype and disease severity are highly variable.

Neonatal hemochromatosis is a clinical condition characterized by severe liver disease in newborns with extrahepatic siderosis^3^. Although most cases of neonatal hemochromatosis are due to gestational alloimmune liver disease, rare associations with congenital disorders such as trisomy 21, mitochondrial depletion syndrome and THES have been reported^3^. However, the molecular mechanism underlying hemochromatosis in THES is unknown. Here, we report a neonatal case of THES with neonatal hemochromatosis, in which novel compound heterozygous variants were identified in the *SKIC3* gene.

A 26-year-old primigravida Japanese woman (I-2 in Fig. 1a) was referred to our hospital owing to fetal growth restriction. She and her husband (I-1 in Fig. 1a) were nonconsanguineous and had no remarkable history. No abnormalities were found on fetal ultrasound examination. Amniotic fluid chromosomal analysis revealed a 46XY karyotype. Fetal ascites appeared at 24 weeks of gestation, and fetal growth arrest was observed from 25 weeks, with a gradual decrease in amniotic fluid volume. Due to persistent fetal growth arrest, an emergency cesarean section was performed at 29 weeks and 6 days of gestation.

**Fig. 1:**
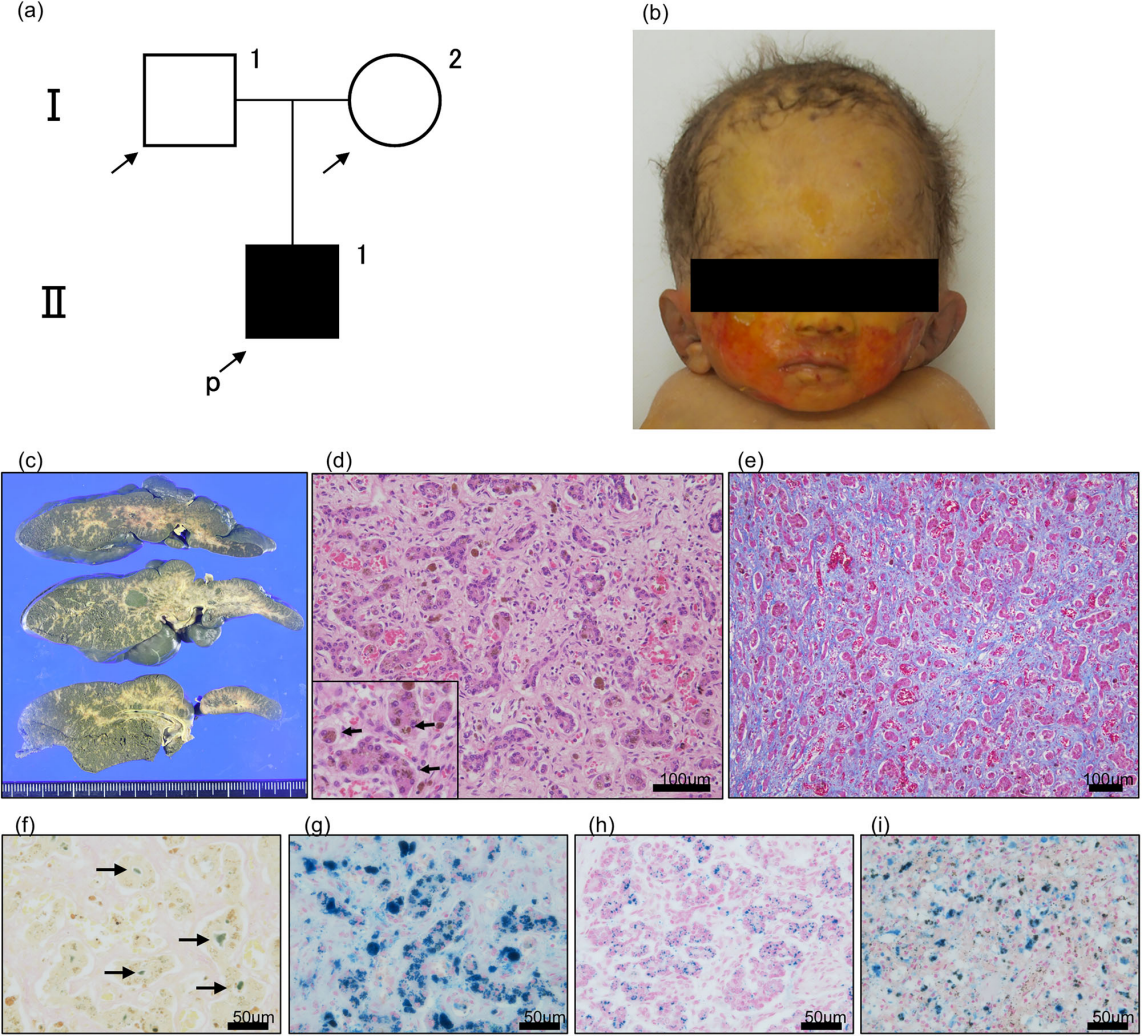
Clinical and pathological findings. **a** Pedigree of the family. **b** Facial photograph showing woolly hair and broad forehead. **c** Macroscopically, the cut surface of the formalin-fixed liver is dark green in color and shows diffuse fibrosis. **d** Hematoxylin and eosin staining shows liver fibrosis and loss of hepatocytes in association with bile-duct proliferation. Dark-brown granular pigmentation is present in many hepatocytes (arrows in inset). **e** Azan staining reveals extensive fibrosis of the liver tissue. **f** Bile stasis is recognized as an emerald-green deposit by Hall’s stain (arrows). **g** Iron staining demonstrates the blue granules of hemosiderin in hepatocytes. **h**, **i** Iron staining of pancreas (**h**) and spleen (**i**) shows hemosiderin deposition.

The infant was male and had a birth weight of 702 g (−3.5 s.d.), birth length of 29.0 cm (−4.2 s.d.) and head circumference of 23.2 cm (−2.1 s.d.). His Apgar scores at 1 and 5 min after birth were 3 and 5, respectively. He was intubated, administered surfactant and admitted to the neonatal intensive care unit. He had a broad forehead and woolly hair (Fig. 1b). The newborn examination revealed severe anemia and hypoglycemia. Ultrasound examination showed intestinal dilatation and minimal ascites. Hypoglycemia improved promptly after the initiation of continuous glucose infusion. The cause of the anemia was not known. Testing for fetomaternal transfusion was negative, and there were no findings suggestive of hemolytic anemia. Several red blood cell transfusions were performed, improving anemia. Due to marked abdominal distension and the absence of meconium passage, an enterostomy was performed at 3 days of age. Intraoperative findings led to a diagnosis of meconium-related ileus. Enteral nutrition was began at 7 days. However, 2 days after initiating enteral nutrition, he developed watery diarrhea. Bilirubin levels continued to rise over time, with direct bilirubin measuring 2.3 mg/dl on day 2, 7.9 mg/dl on day 16 and >10 mg/dl by 30 days of age. Although ascites was minimal at birth, it increased around 3 weeks of age. Abdominal paracentesis was performed at 28 days. Analysis of the ascitic fluid revealed an exudative nature, suggesting an association with liver dysfunction. Ultimately, the patient developed respiratory and circulatory failure due to infection and liver failure and died at 49 days of age.

Autopsy revealed extensive hepatic fibrosis both grossly and histologically (Fig. 1c–e). There was loss of hepatocytes, and ductular reaction was evident (Fig. 1d). Furthermore, many hepatocytes were found to contain dark-brown granular pigmentation, which was identified as blue granules of hemosiderin in iron staining, indicating hemosiderosis (Fig. 1d, g). Bile stasis was observed in the lumen of the bile ducts, recognized as emerald-green deposits using Hall’s stain (Fig. 1f). Hemosiderin deposition was also observed in the pancreas, spleen and thyroid, supporting the diagnosis of neonatal hemochromatosis (Fig. 1h, i). Other lesions included hypoplasia of erythroblastic series in bone marrow and bilateral bronchopneumonia.

A trio whole-exome analysis was performed as described previously^4^. We identified novel compound heterozygous variants in *SKIC3* (OMIM 614589): one frameshift variant with maternal origin, NM_014639.4:c.815_816del p.(Gly272AlafsTer9) in exon 11, and one missense variant with paternal origin, NM_014639.4:c.2284G>A p.(Gly762Arg) in exon 21 (Fig. 2). These variants were confirmed using Sanger sequencing. Neither of the variants has been reported in the Genome Aggregation Database (gnomAD)^5^. The patient exhibited fetal growth restriction, characteristic facial features (woolly hair and broad forehead), refractory diarrhea and liver disease (hemochromatosis), all of which were consistent with the clinical characteristics of THES. According to the guidelines^6^, the maternally derived frameshift variant was classified as ‘pathogenic’ (PVS1: frameshift, PM2: absent from controls, PP4: patient’s phenotype). The missense variant was predicted to be harmful by in silico evaluation (CADD PHRED score 26.1, SIFT score 0, PROVEAN score −5.06 and Mutation taster score 1) and classified as ‘likely pathogenic’ (PM2: absent from controls, PM3: in trans with pathogenic variant, PP3: in silico analysis, PP4: patient’s phenotype).

**Fig. 2:**
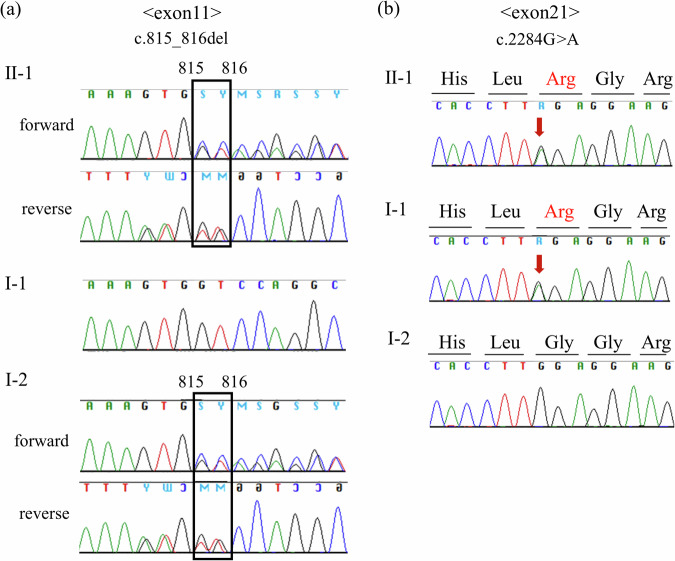
*SKIC3* sequence of the patient and his parents. **a** A frameshift variant, NM_014639.4:c.815_816del p.(Gly272AlafsTer9), in exon 11 in the infant and his mother (I-2). **b** A missense variant, NM_014639.4:c.2284G>A p.(Gly762Arg), in exon 21 in the infant and his father (I-1).

Although the causative genes of THES have been identified, the clinical presentation varies widely, and the relationship between genotype, phenotype and disease severity remains unclear. So far, five cases of THES with hemochromatosis have been reported: four died within 6 months of birth, and the remaining case by 10 months of age^7^. Therefore, THES with hemochromatosis appears to have a poor prognosis. The severe clinical course in this case may have been influenced by both hemochromatosis and extreme prematurity.

No previous cases of THES with hemochromatosis have been confirmed genetically, and the relationship between *SKIC3* function and hemochromatosis remains unknown. *Ski3* (a homolog of human *SKIC3*)-deficient *Drosophila* showed low levels of mitochondrial membrane potential and enzymatic activity in electron transport chain complexes. This evidence indicates that *ski3* deficiency causes mitochondrial dysfunction^8^. Given the critical role of mitochondria in iron metabolism^9^, dysfunction of *SKIC3* may lead to hemochromatosis through mitochondrial impairment.

In conclusion, we report a neonatal case of THES with neonatal hemochromatosis, in which novel compound heterozygous variants in *SKIC3* were identified. Further research, including functional studies, is needed to elucidate the molecular mechanisms linking *SKIC3* to iron metabolism and disease severity in THES.

## HGV Database

The relevant data from this Data Report are hosted at the Human Genome Variation Database at 10.6084/m9.figshare.hgv.351810.6084/m9.figshare.hgv.3521.
